# Dynamic contact networks of patients and MRSA spread in hospitals

**DOI:** 10.1038/s41598-020-66270-9

**Published:** 2020-06-09

**Authors:** Luis E. C. Rocha, Vikramjit Singh, Markus Esch, Tom Lenaerts, Fredrik Liljeros, Anna Thorson

**Affiliations:** 1 Department of Economics, Ghent University, Ghent, Belgium; 2 Department of Physics and Astronomy, Ghent University, Ghent, Belgium; 3 Lidl stiftung & co. KG, Neckarsulm, Germany; 4 Department of Engineering, Saarland University of Applied Sciences, Saarbrücken, Germany; 5 MLG, Université Libre de Bruxelles, Brussels, Belgium; 6 AI-lab, Vrije Universteit Brussel, Brussels, Belgium; 7 Interuniversity Institute for Bioinformatics, Brussels, Belgium; 8 Department of Sociology, Stockholm University, Stockholm, Sweden; 9 Department of Public Health Sciences, Karolinska Institute, Stockholm, Sweden; 10 World Health Organisation, Geneva, Switzerland

**Keywords:** Complex networks, Nonlinear phenomena, Computational science

## Abstract

Methicillin-resistant Staphylococcus aureus (MRSA) is a difficult-to-treat infection. Increasing efforts have been taken to mitigate the epidemics and to avoid potential outbreaks in low endemic settings. Understanding the population dynamics of MRSA is essential to identify the causal mechanisms driving the epidemics and to generalise conclusions to different contexts. Previous studies neglected the temporal structure of contacts between patients and assumed homogeneous behaviour. We developed a high-resolution data-driven contact network model of interactions between 743,182 patients in 485 hospitals during 3,059 days to reproduce the exact contact sequences of the hospital population. Our model captures the exact spatial and temporal human contact behaviour and the dynamics of referrals within and between wards and hospitals at a large scale, revealing highly heterogeneous contact and mobility patterns of individual patients. A simulation exercise of epidemic spread shows that heterogeneous contacts cause the emergence of super-spreader patients, slower than exponential polynomial growth of the prevalence, and fast epidemic spread between wards and hospitals. In our simulated scenarios, screening upon hospital admittance is potentially more effective than reducing infection probability to reduce the final outbreak size. Our findings are useful to understand not only MRSA spread but also other hospital-acquired infections.

## Introduction

The increasing resistance of some bacteria to currently available antibiotics has become a major public health issue in recent decades^1–3^. Hospital-acquired (HA) Methicillin-resistant Staphylococcus aureus (MRSA) infection has been routinely detected in hospitalised patients including those in high-income countries. In the European Union alone 150,000 patients are affected annually^4^. In Sweden, for instance, the incidence rate (per 100,000 people) of MRSA jumped from 10.76 in 2005 to 29.96 in 2014^5^. Although some strains may be harmless to healthy people or are not health-care associated, such difficult-to-treat infections are particularly dangerous in a context of individuals with weakened immune systems^6,7^. A healthy person becomes infected via direct contact with an infected host, or contaminated devices and surfaces^8^. A hospital setting, if not under strict hygienic control, provides excellent conditions for efficient spread of MRSA. Although colonisation typically occurs in the anterior nares, open wounds or intravenous catheters are also potential sites for infection. Therefore, the daily contact between health care workers (HCW) and patients is sufficient for the propagation of the pathogens. This is worsened because colonised individuals, even if not ill, may still infect others. During a regular shift, HCWs typically interact with various patients whereas patients usually interact with different HCWs. These interactions create dynamic contact networks^9,10^ in which the MRSA infection eventually propagates. These contact networks have a complex structure of who was in contact with whom at a given time because of the non-trivial dynamics of a typical day in a hospital^11–14^. Although the transmission between a patient and a HCW is more likely in certain wards (e.g. burns or transplant unit^8^), the mobility of patients between wards or hospitals^15,16^ creates the missing links sustaining the spread of HA-MRSA across the hospitalised population. Therefore, identifying which contact patterns (or network structures^17^) regulate the propagation of the infection is the first step to better understand the spread potential of MRSA, and then to develop efficient strategies to reduce the incidence in endemic areas and to avoid potential outbreaks in low-prevalence contexts^18^.

Mechanistic models aim to generate an abstract mathematical representation of the most relevant characteristics of a system. The gain in understanding, particularly of the causal relations or mechanisms driving the chain of events, by simplifying the problem compensates the information that is discarded. This approach, successfully used in other disciplines for example to forecast the weather or the movement of planets, fundamentally differs from traditional epidemiological methods such as controlled experiments and logistic regression models. A mechanistic model allows assessing realistic scenarios without the need to experiment on the real population. These models in fact have been used for a long time to study the spread of infections at the population level and they are necessarily simplified and focus on key aspects of the contagious dynamics otherwise simulations are not possible^19,20^. In the context of MRSA, a number of studies have been published in recent years mostly using the frameworks of compartmental and agent-based models^21^. Compartmental models are convenient because they can be elegantly described by a set of coupled differential equations, used to describe a group of well-mixed individuals in a certain state^22–24^, and can be sometimes studied analytically^23,25^. Agent-based models, on the other hand, request more computational resources but are more flexible and allow the addition of different levels of complexity by defining updating and interaction rules for each individual^26–28^. Previous models typically focused either on the population dynamics within a single ward^29^ or in a single hospital with a few wards^23,30^, between one hospital and the community^31^, or between multiple hospitals with a simple ward structure^23,27^. As in any modeling exercise, these studies make a series of assumptions regarding different aspects of the population. In particular, exponential probability distributions are defined to account for the patient’s length-of-stay, readmission, or referral to another hospital^22,23,25^. Even in models with some structure, i.e. those that have more than one ward or hospital, average values were used to characterise similar units, as for example, the size of the hospital or the frequency of interactions between HCWs and patients^23,27,32^. In other words, previous models attempted to reproduce the hospital population but failed to fully capture the heterogeneities and complexity of the contact patterns and patient’s mobility, emerging as a consequence of referrals and re-admissions within and between hospitals^33^.

In this paper, we propose a data-driven network model of patients of the Stockholm County in Sweden, covering a population of about 2,192,433 inhabitants. By using information of patient flow (inpatients), we are able to reconstruct, at the individual and daily level, the contacts between all patients in all hospitals and nursing homes of the county. In other words, we are able to trace the exact positions of each patient and identify the potential contacts between them over time. These contacts, mediated by HCW or contaminated objects, are the most likely pathways for the spread of HA-MRSA. We assume that a contact exists between two patients if they have been hospitalised in the same ward at the same time. Contrary to agent-based models, our network model is deterministic and captures the exact temporal and spatial heterogeneities contained in the real-data. This methodology focused on the actual patients naturally captures the real-world contact patterns and thus skips several assumptions on the dynamics of patients as for example length-of-stay, re-admittance, mobility between wards and hospitals, hospital and ward sizes, occupancy levels, etc. Using real-world data is particularly important since, as we demonstrate later on, people are different and contact patterns cannot be simplified by the average behaviour as usually done. For example, long hospital stays increase the risk of infection per stay and may compensate a relatively low risk of transmission per contact, mobility between hospitals and re-hospitalisation play crucial roles to spread the infection across the system and to the community, and differences in the hospital structure (wards) may shape the spread within hospitals.

## Results

### Patient dynamics

**Figure 1:**
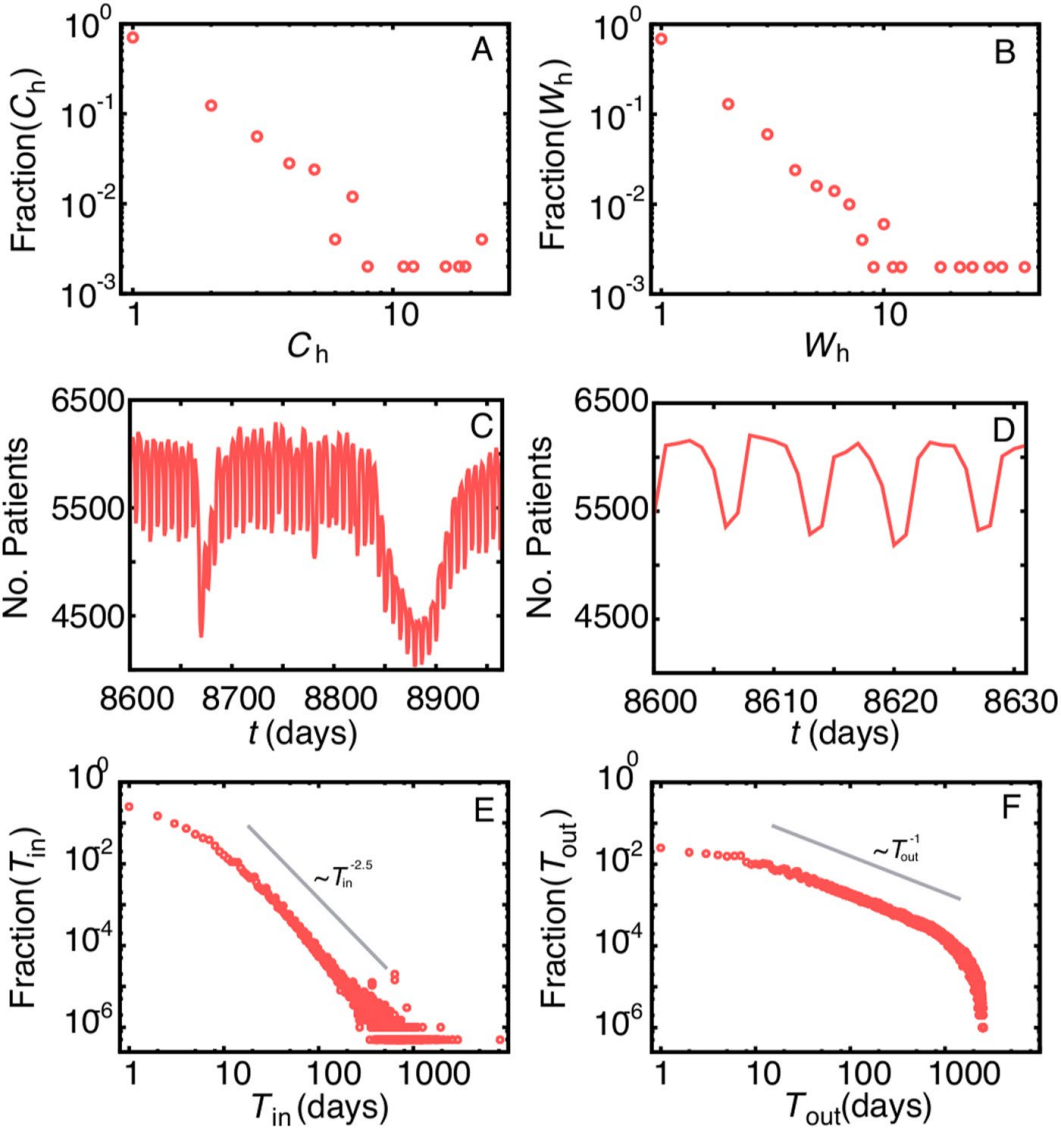
Spatial and temporal patterns. The fraction of hospitals with a given number of (**A**) clinics (*C*_*h*_) or (**B**) wards (*W*_*h*_). Both axes are in log-scale; (**C**) annual and (**D**) weekly cycles of number of inpatients. Times are shifted to anonymise data; Distribution of the (**E**) duration of length-of-stay (*F*(0) = 0.09) and (**F**) times between two subsequent hospitalisations by the same inpatient (*F*(0) = 0). Both axes are in log-scale.

The hospital system contains 859 clinics and 979 wards distributed within 485 hospitals and nursing homes (See methods). We only look at inpatients. There is an asymmetry in which the majority of the hospitals contain a single clinic and a single ward whereas only 7(11) locations contain 10 or more clinics (wards) (Fig. 1A,B). This heterogeneity in the internal structure of hospitals indicates that studying isolated units or making uniform or homogeneous assumptions^23,27,29,30^ inefficiently capture the complexity of the hospital system. While most hospitals are peripheral and potentially less likely to be affected by an epidemic outbreak, larger hospitals behave like hubs with high influx of patients and internal transfers between wards or clinics. The analysis also indicates strong weekly and annual patterns in which the hospital population may decrease by 25% (Fig. 1C) during the summer and winter breaks (when some wards are closed), and about 10% (Fig. 1D) over the weekend in comparison to week-days. At different scales, these cycles may directly affect spreading since the number of new infections correlates with the population size.

The distributions of the length-of-stay in the same ward (*T*_in_) and the times between subsequent hospitalisations (*T*_out_) also follow non-trivial patterns (Fig. 1E,F), strongly differing from the standard homogeneous (i.e. exponential) assumptions. Such particular right-skewed distributions have been observed in human social contacts^34,35^. Within hospitals, for example, proximity interactions (a proxy for transmission of infections) between HCW and patients follow similar distributions^35^. Our analysis show that the vast majority of patients spend a few days in the hospital (80% of the patients spend up to 7 days in the same ward) whereas a substantially reduced number of patients (0.05%) may spend over a year in there (Fig. 1E). Long stays both increase the risk of being infected and of further transmission. In case such long-staying patients become colonised and remain untreated, they act as reservoirs of bacteria. Modeling studies typically assume an average duration of hospitalisation for all patients and thus miss the impact of these long staying individuals in the spread process. The time interval between two hospitalisations shows that 12.7% of the patients are readmitted within a week (30.1% within 30 days) after discharge whereas 26.4% of them return to the hospital more than a year after being discharged (Fig. 1F). The results indicate the nonexistence of a characteristic behaviour and the sharp cut-off in Fig. 1F is due to the finite-size of the data. We also observe that the probability to return to the hospital (re-admission) decreases with the time between two hospitalisations (see SI, Fig. S1A) and that the average hospitalisation times decrease with the time between two hospitalisations (see SI, Fig. S1B). These results indicate that hospitalisations tend to be short but frequent, suggesting high movement of people between the hospital and the community, a dynamics that contributes to the spread of the infection since those patients act as bridges between the different populations. Overall, these temporal correlations show that individuals are highly heterogeneous and do not follow characteristic behaviours, as a consequence, each of them contributes differently to the spread of infections, with some potentially having disproportionate higher importance on the infection spread dynamics.

### Infection dynamics

**Figure 2:**
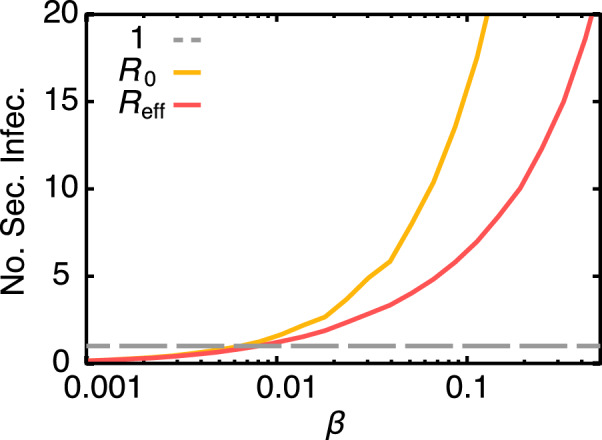
Reproduction number. The relation between the per-contact infection probability *β* and the reproduction numbers *R*_0_ and *R*_eff_ according to simulations of the spread of the MRSA infection. The dashed grey line at 1 represents the epidemic threshold. The x-axis is in log-scale. Standard errors ($=\sigma /\sqrt{n}$, where *σ* is the standard deviation), calculated using *n* = 25,000 realisations of the simulation, are smaller than the thickness of the curves.

To study the population dynamics of MRSA, we select one year of the original data set and extract the exact contact patterns between the patients. This sample has 170,839 patients and 20,499,964 contacts. For simplicity, we assume that newly admitted patients are not colonised or infectious (i.e. *α*_adm_ = 0) and no treatment is available. We also assume that *β*_C_ = *β*_I_ = *β* and then scan the values of the per-contact infection probability *β* (our model naturally captures the real-world contacts over time and thus contact rates are meaningless; we use instead the per-contact infection probability) to estimate the basic and effective reproduction numbers (*R*_0_ and *R*_eff_ respectively). The reproduction number (also known as the number of secondary infections) defines a threshold in which a large epidemic outbreak may occur if *R*_0_ > 1 (or *R*_eff_ > 1). In highly clustered populations like the one studied here, local depletion of susceptible patients occurs and thus the effective reproduction number better characterises the epidemic threshold than the basic reproduction number. To estimate *R*_0_, we first infect a single individual (at different times) and set all other individuals as susceptible; then we simulate the spread of the infection and count the number of secondary infections made by the seed infected individual until its recover. We repeat this procedure 25,000 times with different starting individuals to calculate the statistics (here and elsewhere, results are quantitatively similar if we consider 50,000 realisations of the simulations). The estimation of *R*_eff_ is similar to *R*_0_ with the difference that already infected individuals are not taken into account when counting the number of secondary infections, i.e. it is not possible to re-infect already infected individuals as normally done on calculations of *R*_0_. For example, consider the simple case of node A connected to B and C, with B also connected to C. Assume that the infection starts at A, A infects B and B infects C; if later on, A (re-)infects C, this infection event A-C is not counted on *R*_eff_ but is counted for *R*_0_. Figure 2 shows that the epidemic threshold for *R*_eff_ is at *β* ~ 0.008 (as expected, this threshold is slightly lower for *R*_0_, i.e. *β* ~ 0.007), implying that if the per-contact infection probability is larger than *β* ~ 0.008, a large epidemic outbreak is likely to happen. The effect of the high clustering (i.e. depletion of contacts) is stronger for larger infection probabilities, e.g. *R*_0_ ~ 3*R*_eff_ for *β* = 0.1, because the infection spreads faster within close contacts.

**Figure 3:**
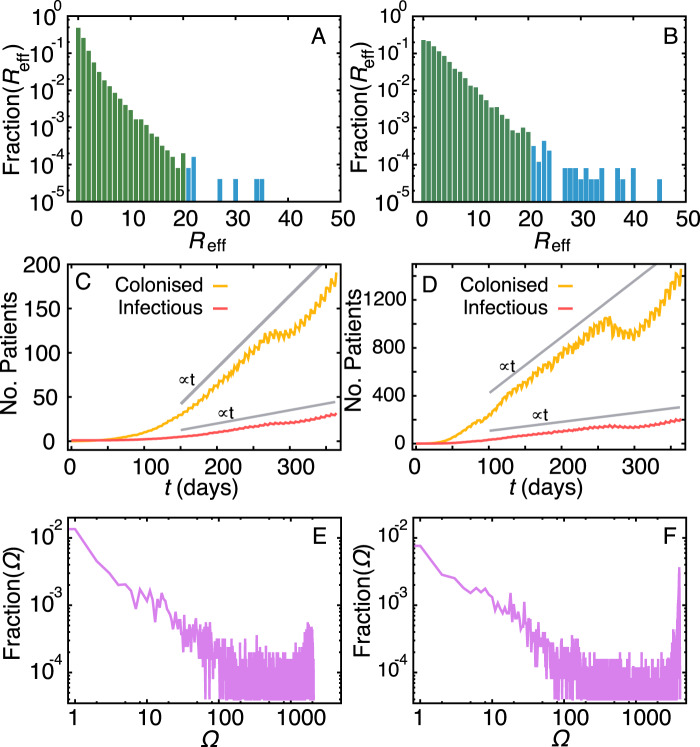
Evolution of the state of patients. Distribution of the effective reproduction number *R*_eff_ for (**A**) *β* = 0.01 and (**B**) *β* = 0.03. Values above 20 are highlighted in blue. y-axes are in log-scale. The evolution of the number of colonised and infectious individuals for (**C**) *β* = 0.01 and (**D**) *β* = 0.03. Straight lines (∝*t*) are draw for guidance. The distributions of final outbreak sizes (i.e. number of colonised plus infectious at *T* = 365 days) for (**E**) *β* = 0.01 and (**F**) *β* = 0.03. Null outbreaks are given by *P*_0.01_(0) = 0.76 and *P*_0.03_(0) = 0.47. Both axes are in log-scale.

The heterogeneity of the network structure implies that patients do not have the same importance in the transmission dynamics. Looking at the distribution of *R*_eff_, i.e. the distribution of the number of secondary infections when we start the infection at random patients, we observe that for both low (*β* = 0.01) and high (*β* = 0.03) infection probabilities (chosen because they are both just above the estimated threshold), most patients cause zero or a few secondary infections whereas a very few patients may cause more than 20 secondary infections (Fig. 3A,B), which may characterise them as super-spreaders^36^ given that $\langle {R}_{{\rm{eff}}}\rangle =1.22$ and the variance *σ*^2^ = 4.04 (for *β* = 0.01) and $\langle {R}_{{\rm{eff}}}\rangle =2.78$ and *σ*^2^ = 10.07 (for *β* = 0.03). This is in contrast to well-mixed models (e.g.^24^) and is a consequence of a patient making too many contacts over a period of time, that is not necessarily connected to the length-of-stay (See SI, Fig. S2A–D).

We investigate the growth curve of the epidemic outbreak for the same two values of the per-contact infection probability (Fig. 3C,D). It takes around 100 days for the number of infectious individuals to take off, yet, in both cases, the curves are characterised by a (trend) linear growth (∝*t*) after the initial low-prevalence regime (Fig. 3C,D). This is likely a combined effect of adding new patients to wards and the heterogeneous length-of-stay of patients. The effect of the lower number of patients during holidays appears at around 250 days after the onset of the epidemics (the location of this effect depends on the starting dates of the simulation), however, the decrease in the number of colonised and infectious individuals is not as significant as the decrease in the total number of patients (compare to Fig. 1C). The weekly pattern modulates but does not affect the trend of the number of colonised and infectious individuals. The distribution of the final outbreak sizes Ω (i.e. the number of colonised plus infectious individuals at *T* = 365 days) has a bi-modal shape (Fig. 3E,F). It indicates that a large number of outbreaks will cause none or a few infections (83.2% and 52.3% –for *β* = 0.01 and *β* = 0.03 respectively– of the outbreaks generate less than 100 infections) but there is also a significant chance of large outbreaks affecting more than 1000 individuals (11.8% and 43.9% for *β* = 0.01 and *β* = 0.03 respectively). This bi-modal shape results from a combination of the heterogeneity in the duration and in the number of contacts^37,38^.

### System-wide spread

**Figure 4:**
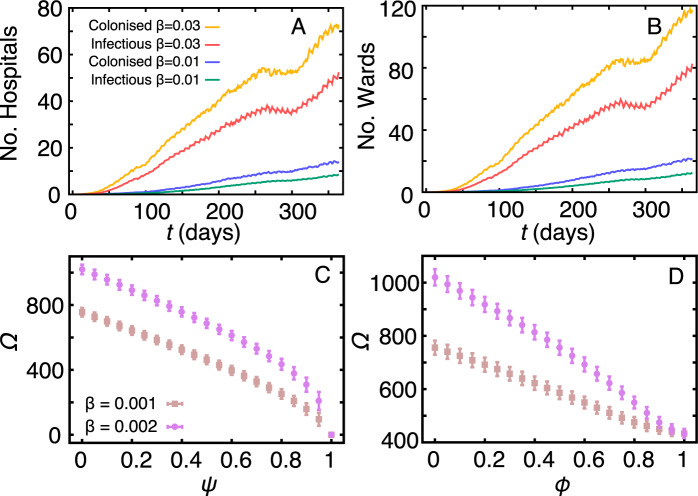
Prevalence on hospitals and wards, and infection control. The evolution of the number of colonised and infectious (**A**) hospitals and (**B**) wards. A hospital or ward is colonised or infectious if it contains at least one colonised or one infectious patient, respectively; (**C**) the outbreak size Ω vs. screening level during patient admission *ψ*; (**D**) the outbreak size Ω vs. hygienic level *ϕ*. In both cases, the final outbreak size Ω is calculated as the average number of colonised plus infectious individuals during the last week of the simulation, i.e. during *T* = [359, 365] days.

We follow the number of hospitals and wards with at least one colonised or one infectious individual to study the infection spread between hospitals and wards. We observe that in our sample, with 306 hospitals and 521 wards, the number of infected hospitals increases 5 times (and infected wards increase 8 times) one year after patient zero, if we increase the infection probability from *β* = 0.01 to *β* = 0.03 (Fig. 4A,B). For low infection probabilities (*β* = 0.01), very few hospitals and wards are infectious (or colonised) within 100 days since the onset of the epidemics. A similar epidemic scenario (i.e. prevalence) is observed within about 40 days in case of *β* = 0.03. On average, the first observed colonised and infectious case outside the hospital in which the epidemic started happens respectively at time $\langle {T}_{{\rm{exit}}}\rangle =14.33$ (*σ*^2^ = 375.61) and $\langle {T}_{{\rm{exit}}}\rangle =24.76$ (*σ*^2^ = 1010.25) days for *β* = 0.01. For *β* = 0.03, the situation is more critical and colonised and infectious cases occur respectively at $\langle {T}_{{\rm{exit}}}\rangle =10.21$ (*σ*^2^ = 198.22) and $\langle {T}_{{\rm{exit}}}\rangle =18.89$ (*σ*^2^ = 604.21) days. These results indicate that in conservative scenarios, it takes 3.5 weeks for the infection to appear in a second hospital (either because of transfer of an infected patient or because a colonised patient develops infection there). In the most critical scenario, it takes only about 2.5 weeks. Results are similar if we look at the epidemic spread between the source ward and a second ward (instead of between hospitals), except that the infection spreads slightly faster. This is expected because most locations contain a single ward (see Fig. 1B).

### Infection control

We study the effect of simulated screening and hygienic in the infection dynamics. In this experiment, we assume that newly admitted patients may be colonised with probability *α*_adm_ = 0.0877^29^, implying on a constant influx of colonised patients that is different from the assumption of the previous sections. This assumption accounts well for a conservative worst-case scenario and should not be taken as the current colonised admission rate in Sweden. We also assume that infectious and screened colonised patients undergo treatment, lasting on average *τ*_treat_ = 7 days. During treatment, patients are quarantined and cannot infect or be re-infected. To simulate screening, we select a random fraction *ψ* of the admitted patients and put them in treatment if colonised; susceptible patients have no special protocol. For hygienic, we tune the per-contact infection probability by a factor 1 − *ϕ* (i.e. higher *ϕ* means higher hygienic as for example increasing hand washing), that is, we set the per-contact infection probability to *β*(1 − *ϕ*).

In contrast to previous sections, the daily influx of colonised patients now implies that a lower per-contact infection probability is sufficient to sustain the epidemics. We thus test two scenarios: *β* = 0.001 (low) and *β* = 0.002 (high), i.e. both bellow the epidemic threshold. Figure 4C shows that for both infection probabilities, increasing the fraction of screened patients decreases the final outbreak size Ω. Screening 50% of the admitted patients reduces the final outbreak by 330025–38% in comparison to no screening. Independently of *β*, full screening is necessary to reduce Ω to zero. Figure 4D shows that reducing the infection probability by 50% reduces by 22–26% the final outbreak size (measured in the same way as for the screening) in comparison to no action. Here, null per-contact infection probability (*ϕ* = 1) may still result in a few cases since some patients may be contaminated when admitted. It is difficult to directly compare both strategies because different than screening, there is no one-to-one correspondence between hygienics and *ϕ*, given that the last involves not only compliance on washing hands or using gloves but also on cleaning of rooms and utensils.

## Discussion

Antibiotic-resistant bacteria have been increasingly pressing hospital systems in both low and high-income countries. Methicillin-resistant Staphylococcus aureus (MRSA), in particular, is difficult to treat and can be particularly dangerous to people with weakened immune system such as hospitalised individuals. Although antibiotic-resistance has developed due to intense use of antibiotics, MRSA commonly spreads through physical contacts with infected individuals, objects, or surfaces. Hygienics is generally argued as effective means to avoid the propagation of MRSA^3,8^ but enforcing such routine in daily practice among HCWs remains challenging mostly due to socio-economic factors. Not least, recent studies point out to other factors potentially affecting spreading not only of MRSA but also of other resistant pathogens^39^. Such scenario calls for a better understanding of the population dynamics of such infections and modelling studies contribute to experiment hypotheses without interfering in the study population.

To understand the dynamics of MRSA infections, we developed a realistic data-driven model of contact patterns between 743,182 inpatients in a large hospital system in Sweden based on the exact location of patients over time. Our innovative model captures the exact complex spatial and temporal heterogeneities (inherent to the structure of hospitals) and patients’ behaviour (e.g. referrals, length-of-stays) into a dynamic contact network structure. We first study these interactions and hospitalisation dynamics during 8 years to identify non-trivial temporal and mobility patterns potentially affecting the spread of hospital-acquired infections in hospitals. We then couple a MRSA contagion model on this population to experiment worst-case scenarios and test how individual behaviour affects spreading. Our simulations indicate that the epidemic threshold (*R*_eff_ = 1) is reached if *β* ~ 0.008. Nevertheless, with a constant influx of colonised individuals, as typically occur in the real-world, an epidemic may be sustained with lower per-contact infection probabilities. If we set the entire population susceptible and a single patient as infected, we observe that right above the epidemic threshold, the epidemic curve grows linearly after an initial low-prevalence period lasting about 100 days. This is a consequence of the heterogeneities in the contact patterns that slowdown the spread and refrain an exponential growth, typically observed in theoretical epidemic models. Linear growth is rarely observed in theoretical models^40^, possibly because of disregarding dynamic heterogeneous contact patterns^37^. A detailed analysis requests further analysis of the models and temporal patterns. Another consequence of patient’s behaviour is the bi-modal distribution of outbreak sizes in which the likelihood of minor outbreaks (<100 infected individuals) is relatively high, but larger outbreaks (>1000 individuals) are also common (with intermediate cases less likely). This is in accordance with previous reports of MRSA outbreaks in Sweden during the 1990s^18^. We also observe the appearance of a few super-spreaders, i.e. individuals infecting a much larger than average number of patients. Super-spreading in this context correlates more with the number of contacts per patient than with length-of-stay, suggesting that long-term patients should stay together to avoid high patient turnover in shared rooms. In our tested scenarios, the simulated epidemics can spread relatively fast, taking only 2 to 4 weeks to reach a second hospital or ward. Even for low infectious rates, MRSA may reach up to 10 hospitals within a year after the initial infection. These results emphasise not only the need of quick responses (e.g. quarantine and treatment) once a positive case is detected but also quick testing of suspect cases.

One of the goals of modeling exercises is to understand the mechanisms sustaining the spread of the infection to reduce its incidence and hence mortality and costs. The analysis of the population dynamics is often done by comparing the implementation of some infection control protocol against the absence of any action. In our experiments, we show that reducing the per-contact infection probability by 50%, as a consequence of improved hygienic (e.g. washing hands or better room cleaning) is not sufficient to half the final outbreak size. Since sufficient cleaning of hands and utensils are many times not achievable in practice, other strategies involving screening followed by isolation and treatment of colonised or infected people have to be introduced. As we have shown, screening every admitted patient is a priori the only way to identify all colonised and avoid an epidemics but this strategy is associated to high financial costs^41^. It is also unrealistic to quarantine every new patient before confirmation of infection, implying that transmission may occur meanwhile. Screening of intensive care patients, at risk wards, or previously documented carriers, on the other hand, have been suggested as alternatives to global screening^28,41^. Since hospitals are strongly connected through transfer of patients, a surveillance system based on a few sentinel hospitals, chosen according to their centrality in this referral network, may be also effective means for early detection, and thus control, of MRSA outbreaks^42^. An agreement on the best cost-effective policies is however still missing.

Our modelling exercise contains limitations in the contagion model to make simulations computationally feasible and also to accommodate the unavailability of data. The main limitation is the assumption that the infection probability is independent of ward type. This information was not disclosed in our data set. It is known however that some wards carry more risk than others and thus infection is more concentrated on patients and HCWs on these wards. While the addition of this information would likely affect numerical estimations, the advantage of our assumption is to single out the effect of contact patterns on the spread dynamics. For example, we were able to identify that super-spreaders exist simply because of heterogeneous contact patterns and not because of higher than average individual risk of infection, that in turn may boost super-spreading. In a more realistic setting, higher risk of infection could potentialise super-spreading. Sweden is a low prevalence setting for MRSA and thus some parameters used in the simulation exercise, obtained from other high-income countries, may be unrealistic for Sweden. Nevertheless, they provide a baseline for worst-case scenarios in a completely susceptible population as we simulate. Parameters estimation in this study, therefore, should only be used to understand the spread mechanisms under hypothetical scenarios and not for public health policy.

The main contributions of our study are the identification of highly heterogeneous contact patterns and patient mobility between hospitals, and how these patterns result on potential super-spreaders, fast spread across wards and hospitals but relatively slow epidemic growth across the system, that is further regulated by weekly and seasonal hospitalisation patterns. Hospitalised populations are in constant movement and infection paths thus depend on both timings and number of interactions, variables neither captured in standard regression and network models, nor cohort and longitudinal studies. Such findings are relevant not only for MRSA but also for other hospital-acquired infections as for example *Klebsiella pneumoniae* or *Clostridium difficile*^43^ that spreads through close contact not necessarily physical, where hand hygiene may not be sufficient to control spreading. The incidence of MRSA has decreased in recent years and some countries, such as Sweden, have managed to keep a very low prevalence. Nevertheless, similar mechanisms are expected to regulate the spread of other HA infectious diseases and our approach based on deterministic real-world temporal contact networks helps to better understand the general phenomena of spatio-temporal infection spread within and between hospitals. A holistic mechanistic perspective, as proposed in our study, involving realistic modelling of complex dynamic human interactions is fundamental to understand the spread and to design strategies to mitigate the epidemics of HA infectious diseases. Our modeling also allows detailed tracing of the potential infection paths in case of an outbreak and can be used to identify hospitals and wards at risk. Such information can be used to develop an efficient sentinel system based on the flow of patients and infections in which all patients at sentinel hospitals or wards are screened and their previous hospitalisations traced back. Our methodology to estimate infection paths allows the identification of source of infections at the individual patient or ward levels. This information could be useful to understand the causes of an epidemic outbreak, improve hygienic and screening, and monitor the behaviour of HCWs. Further modeling improvements could be achieved by considering the variability of infection rates at different wards and the interactions between HCWs and patients.

## Materials and methods

### Patient flow data set

We gather data on the admission and discharge dates of 743,182 inpatients in 485 hospitals and nursing homes at 52 geographical locations in the Stockholm County, Sweden. This information is collected during 3,059 continuous days in the 2000s (the exact years are confidential for ethical reasons). A total of 2,019,236 admissions are recorded. Each patient and the respective ward of hospitalisation are anonymous but have unique IDs for identification.

The data set was not generated by our team. The original data can be obtained by requesting access to the Stockholm County Council (Stockholm läns landsting, www.sll.se) in Sweden, that is a public institution. The Ethical Review Board in Stockholm approved the use of the data (Record 2006/3:3) for our research. The data set contains anonymised information and cannot be linked back to specific individuals. All methods were carried out in accordance with relevant guidelines and regulations.

### Contact network model

**Figure 5:**
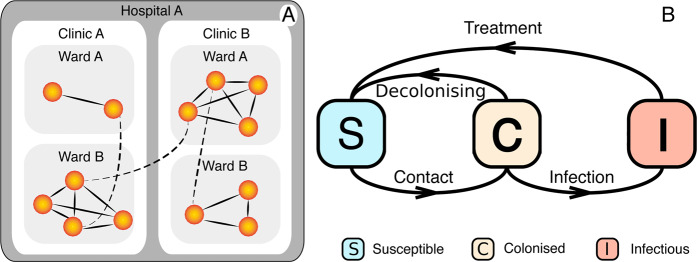
Architecture of the hospitalised population and the MRSA contagion model. (**A**) shows the structure of the contact network within the various levels (wards within clinics, and clinics within hospitals, as available from the real-data) of the Swedish hospital system. A hospital may have one or more clinics, and each clinic may have one or more wards. Nodes, representing the patients, are connected by lines if they have been hospitalised at the same ward at the same time (i.e. the same day). To illustrate the movement between wards, full lines represent contacts at a given time and dashed lines represent contacts made at previous times; (**B**) shows the potential transitions between states in the MRSA infectious disease model. Each inpatient can be either susceptible, or colonised, or infectious at a given day.

A contact network is a set of links connecting pairs of nodes^9,10^. Our data-driven contact network model is formed by inpatients (the nodes) that shared a ward at the same time (Fig. 5A), that is, we connect pairs of inpatients that were at the same ward at the same day. We assume that patient-to-patient contacts occur through HCWs or contaminated objects and surfaces. The time resolution is one day, implying that the contact network changes in time, i.e. a link (representing the contact) between two individuals may exist or not at a given time^9,10^. After each day, links may appear, disappear or be maintained according to the real-world location and dynamics of patients. This is a deterministic contact network model that captures the exact spatio-temporal dynamics where contacts are formed directly from real-world data, it is different from previous agent-based models using stochastic networks^44,45^. Since our network model captures the interaction patterns at high spatial and temporal resolutions, assumptions regarding the structure of the hospitals, mobility, contact rates, or sampling from distribution are unnecessary.

### Contagion model

We devise a novel model capturing the main mechanisms of the MRSA contagion dynamics on the empirical networked population. Our model and parameters are based on the literature and adapted to the unique network structure available in our study^22,24,30^. The model is naturally a simplification of the infection dynamics, yet keeping the key features, to make simulations feasible given the complexity of the infection process and the size of the study population. We thus assume that a single strain circulates in this population and that a patient may be either Susceptible (S), Colonised (C), or Infectious (I)^24^. This is an extension of the standard SIS compartmental model with an extra state C for asymptotic colonised patients. Transitions between these states occur according to the interaction dynamics over the network or to the progression of the infection (Fig. 5B). A susceptible patient can be infected upon contact, with per-contact infection probability *β*_C_ and *β*_I_ (note that it is meaningless to define an infection rate in our model since our real contact network already captures the contacts over time and thus the heterogeneity of contact patterns^37^, in contrast to average contact rates used in stochastic models), from a patient that is C or I, respectively. An individual in the states C or I cannot be re-infected. Although both states C and I are contagious, they differ in the recovery probabilities. Upon infection, i.e. when turning to colonised state C, the patient will further develop the infection with probability *μ* = 0.2^24^ or decolonise with 1 − *μ*. If the patient develops infection, it moves from state C to I after *τ*_infec_ = 9.5 days^24,30^. On the other hand, if the patient decolonises, it moves from state C to S after an average of *τ*_rec_ = 370 days^22^. We use conservative values from the literature to study worst-case scenarios since a full sensitive analysis is computationally unrealistic. We assume that a newly admitted patient may be colonised with probability *α*_adm_ and thus susceptible with 1 − *α*_adm_. If treatment occurs, the patient is cured after *τ*_treat_ and then can be re-infected. We also assume antibiotic resistance does not emerge spontaneously during the study period and discharged patients are assumed to be susceptible. Table 1 summarises the few parameters of the model.

**Table 1:** Parameters of the contagion model.

Parameter	Value
Per-contact Infection probability if C	*β*_C_
Per-contact Infection probability if I	*β*_I_
Probability to develop infection	*μ* = 0.2
Prob. admitted infected	*α*_adm_
Time at stage C if developing infec.	*τ*_infec_ = 9.5 days
Time at stage C if not developing infec.	*τ*_rec_ = 370 days
Time to cure if treated	*τ*_treat_ = 7 days

## Supplementary information

Supplementary Information.
