# Supplementary material for: Dynamic contact networks of patients and MRSA spread in hospitals
Source: Sci Rep. 2020 Jun 9;10:9336. doi: 10.1038/s41598-020-66270-9 (PMC7283340; doi:10.1038/s41598-020-66270-9)
Supplement: Supplementary file 1 — Supplementary Information. [file 41598_2020_66270_MOESM1_ESM.pdf]

Supplementary Information: Dynamic contact networks of patients and MRSA spread in hospitals

Luis E C Rocha*

Department of Economics & Department of Physics and Astronomy, Ghent University, Ghent, Belgium

Vikramjit Singh

Lidl stiftung & co. KG, Neckarsulm, Germany

Markus Esch

HTW Saar, Saarbrücken, Germany

Tom Lenaerts

Université Libre de Bruxelles, Brussels, Belgium

Fredrik Liljeros

Department of Sociology, Stockholm University, Stockholm, Sweden

Anna Thorson

Karolinska Institute, Stockholm, Sweden & World Health Organisation, Geneva, Switzerland

(Dated: May 4, 2020)

I. Patient Dynamics

Supplementary Figure S1A shows the return probability, i.e. the chance of returning to the hospital, for a given period of time outside the hospital. We see that this probability increases slightly between 1 and 10 days outside the hospital, and then decreases substantially for more than 10 days. This indicates a relatively high chance of returning to the hospital within 10 days after discharge. Supplementary Figure S1B shows the hospitalization length after being outside the hospital for a given duration of time. There is a small decrease in the hospitalization length for increasing time outside the hospital. This means that one expects longer hospitalization times within few days after discharge.

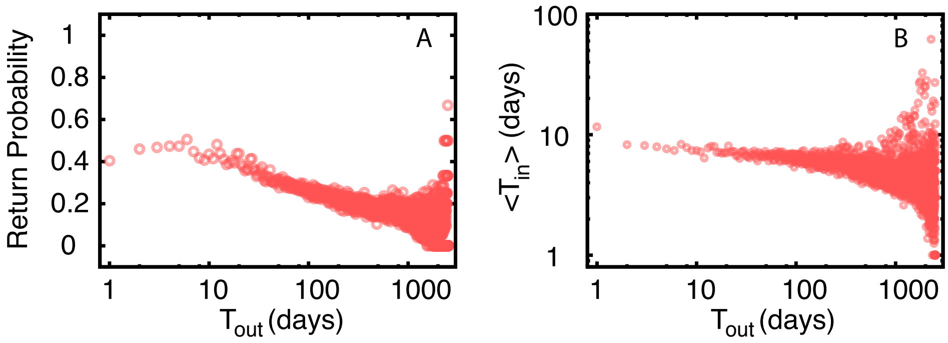

FIG. 1: **Return probability and hospitalization length.** (A) shows the return probability vs. the time outside the hospital. x-axis in log-scale; (B) shows the average hospitalization duration vs. the time outside the hospital just before hospitalization. Axes in log-scale.

*Electronic address: luis.rocha@ugent.be

II. Reproduction Number

Supplementary Figures S2A,C show the correlation between the effective reproduction number and the number of contacts for a given individual. We see a strong dependence for both infection probabilities. Supplementary Figures S2B,D show the correlation between the effective reproduction number and the hospitalization length for a given individual. We see that there are positive dependences but they are not much strong.

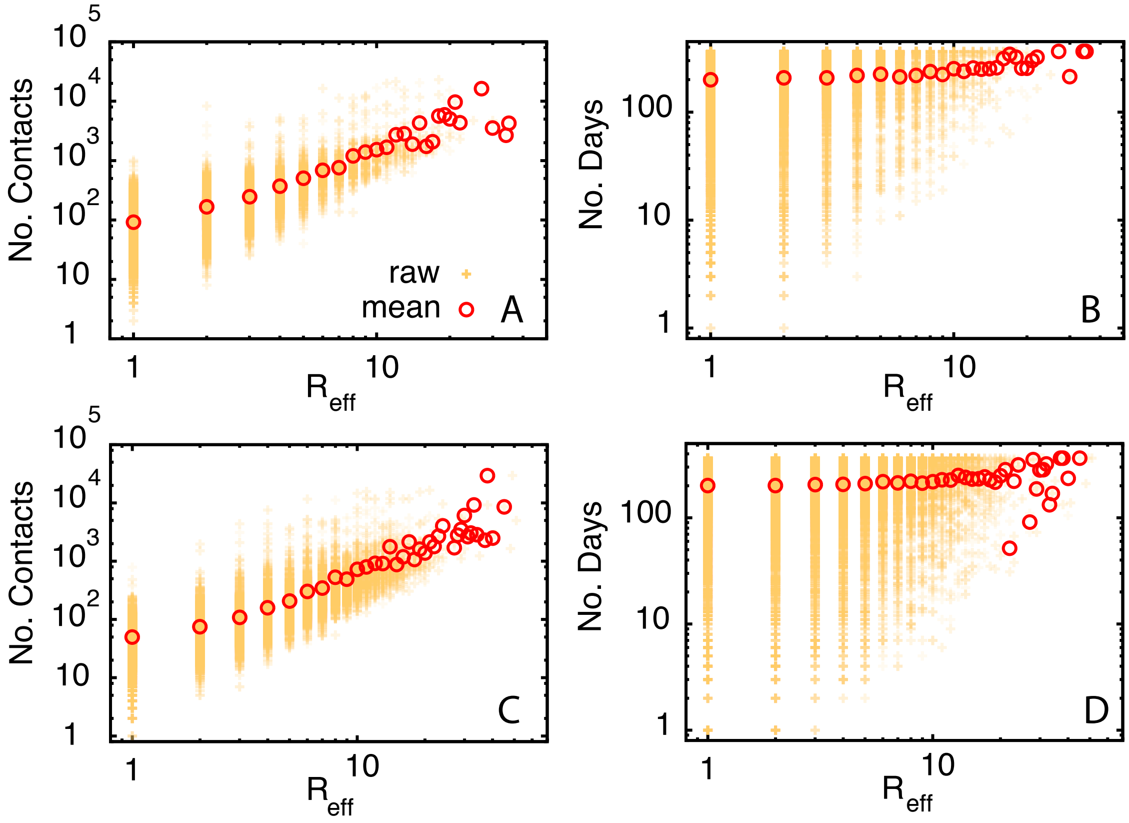

FIG. 2: **Reproduction number, contacts and duration of hospitalization.** Correlation between the individual effective reproduction number (R_{eff}) and the individual number of contacts for (A) $\beta = 0.01$ and (C) $\beta = 0.03$; Correlation between the individual effective reproduction number (R_{eff}) and the duration of hospitalization for (B) $\beta = 0.01$ and (D) $\beta = 0.03$. Each plot contains 25000 points and the mean values for a given R_{eff}. The axes are in log-scale.
